# *miR-449a*: A Novel Biomarker for Diagnosis, Prognosis, and Treatment Response in Locally Advanced Laryngeal Squamous Cell Carcinoma

**DOI:** 10.32604/or.2025.073051

**Published:** 2026-02-24

**Authors:** Amal F. Gharib, Ohud Alsalmi, Hayaa M. Alhuthali, Afaf Alharthi, Saud Ayed Alharthi, Shaimaa A. Alharthi, Rasha L. Etewa, Wael H. Elsawy

**Affiliations:** 1 Department of Clinical Laboratory Sciences, College of Applied Medical Sciences, Taif University, P.O. Box 11099, Taif, 21944, Saudi Arabia; 2 Department of Otorhinolaryngology, King Abdulaziz Specialized Hospital, Taif, 26521, Saudi Arabia; 3 Department of Medicine, Fakeeh College for Medical Sciences, P.O. Box 2537, Jeddah, 21461, Saudi Arabia; 4 Pathology Department, College of Medicine, Jouf University, Sakaka, 72388, Saudi Arabia; 5 Clinical Oncology Department, Faculty of Medicine, Zagazig University, Zagazig, 44519, Egypt

**Keywords:** Locally advanced laryngeal squamous cell carcinoma (LA-LSCC), *microRNA-449a* (*miR-449a*), biomarker, prognosis, diagnosis, treatment response, serum biomarker, chemoradiotherapy, organ preservation

## Abstract

**Background:**

Locally advanced laryngeal squamous cell carcinoma (LA-LSCC) presents clinical challenges due to the lack of reliable non-invasive biomarkers. This study aimed to evaluate *miR-449a* as a diagnostic and prognostic biomarker in LA-LSCC. Methods: *miR-449a* expression was analyzed in tumor tissues, adjacent normal tissues, and serum from 81 LA-LSCC patients and 50 controls using quantitative real-time reverse transcription polymerase chain reaction (qRT-PCR). We assessed the diagnostic accuracy by Receiver Operating Characteristic curve (ROC curves), clinicopathological associations, survival outcomes (Kaplan-Meier), and treatment response dynamics.

**Results:**

*miR-449a* was significantly downregulated in LA-LSCC tissues (*p* < 0.0001) and serum (*p* < 0.0001), with a strong tissue-serum correlation (R^2^ = 0.988). Tissue *miR-449a* demonstrated a diagnostic accuracy (Area Under the Curve, AUC = 0.857), while serum showed moderate accuracy (AUC = 0.734). High *miR-449a* expression correlated with favorable clinicopathological features and improved survival (median overall survival: 67.82 vs. 23.74 months; *p* = 0.0012). Multivariate analysis confirmed *miR-449a* as an independent prognostic factor (*p* < 0.001). *miR-449a* levels increased post-treatment, particularly in responders to chemotherapy/radiation (*p* < 0.0001).

**Conclusion:**

*miR-449a* serves as a non-invasive biomarker for LA-LSCC diagnosis, prognosis, and treatment monitoring. Its dynamic expression highlights potential for risk stratification and therapy response prediction, warranting further validation in larger cohorts.

## 1 Introduction

Laryngeal squamous cell carcinoma (LSCC) is the most common malignancy of the larynx, accounting for approximately 95% of cases [1]. Locally advanced LSCC (LA-LSCC) poses a significant clinical challenge due to a higher risk of treatment failure compared with earlier stages, greater functional morbidity—particularly affecting phonation, deglutition, and respiration—and a less favorable prognosis with reduced survival rates [2,3].

The strategy of using induction chemotherapy followed by definitive chemoradiotherapy inLA-LSCC is predicated on several key rationales [4]. Induction chemotherapy, typically administered prior to the main locoregional treatment, aims to achieve tumor downstaging, eradicate micrometastatic disease, and assess the tumor’s chemosensitivity [5]. Potential advantages of this approach include an increased likelihood of organ preservation by reducing tumor bulk, potentially enhancing the efficacy of subsequent chemoradiotherapy by improving tumor oxygenation and reducing disease burden [6]. Furthermore, by identifying chemosensitive tumors early, induction chemotherapy may help to select patients who are more likely to benefit from further aggressive treatment, while potentially sparing less responsive patients from unnecessary toxicities associated with definitive chemoradiotherapy alone [7,8]. While its impact on overall survival remains a subject of ongoing research, this sequential approach seeks to optimize locoregional control and improve functional outcomes in this challenging patient population [7].

MicroRNA-449a (*miR-449a*) is a small non-coding RNA with a significant role in cancer. Primarily a tumor suppressor, it targets oncogenes, thereby inhibiting cell proliferation, migration, invasion, and promoting apoptosis by modulating pathways involving genes like *CDK6*, *E2F3*, *ZEB1*, and *Snail* [9]. Downregulation of *miR-449a* is common in cancers like prostate, lung, gastric, and laryngeal, correlating with aggressive behavior and poor prognosis [10]. However, it can have oncogenic roles in some contexts, highlighting its context-dependent function [11–13]. A detailed understanding its specific targets and signaling pathways is crucial for its potential as a diagnostic and therapeutic tool.

A growing body of evidence confirms the tumor-suppressive role of *miR-449a* across various squamous cell carcinomas. Its downregulation has been documented in head and neck SCC (HNSCC), where it is recognized as a clinically promising biomarker [14]. Functionally, in non-small cell lung cancer, *miR-449a* inhibits migration and invasion by directly targeting the c-Met oncogene [15]. Similarly, in esophageal SCC, downregulation of *miR-449a-5p* promotes cell proliferation via dysregulation of cyclin D1 [16]. Most recently, a study specifically in laryngeal SCC demonstrated that miR-449a antagonizes the epithelial-mesenchymal transition (EMT) through IL-6-mediated trans-signaling [17].

Building upon this foundational work, we specifically evaluated its diagnostic utility by analyzing its expression profiles to discriminate between patients with LSCC and healthy control subjects. Furthermore, we explored the potential prognostic significance of *miR-449a* expression levels, examining their correlation with pertinent clinicopathological features and patient outcomes. Through a comprehensive evaluation of *miR-449a* expression patterns, this study aims to validate its suitability as a non-invasive biomarker for LSCC and to assess its capacity to yield valuable prognostic information that could contribute to refined risk stratification and personalized treatment strategies.

## 2 Patients and Methods

### 2.1 Study Population

This prospective study enrolled 81 patients diagnosed with LA-LSCC and 50 healthy control subjects between April 2016 and November 2023. The cohort consisted of 63 males (77.8%) and 18 females (22.2%), with a median age of the patients was 55 years, with an age range of 36 to 69 years. With regard to performance status, 23 patients (28.4%) had an Eastern Cooperative Oncology Group (ECOG) score of 0, 42 patients (51.9%) had a score of 1, and 16 patients (19.7%) had a score of 2. In terms of smoking history, 57 patients (70.4%) were current smokers, while 24 patients (29.6%) were non-smokers. This study was approved by the Ethics Committee of the Faculty of Medicine at Zagazig University (Approval No.: 2016-April-213) and was conducted in accordance with the Declaration of Helsinki. Written informed consent was obtained from all participants, including healthy controls recruited from a routine health screening clinic, prior to their inclusion in the study and any sample collection.

### 2.2 Eligibility Criteria

Patients were eligible for inclusion if they met the following criteria:

1. No prior treatment for LSCC.
2. Histopathological confirmation of LSCC via biopsy.
3. Disease classified as T3 or T4 with nodal status N0, N1, or N2, according to the American Joint Committee on Cancer (AJCC) Staging System [18].
4. An Eastern Cooperative Oncology Group (ECOG) performance status of less than 2 [19].
5. Normal liver, kidney, cardiac, and bone marrow functions, as assessed by standard clinical and laboratory parameters.
6. Adequate nutritional status and absence of significant hearing impairment.
7. Age < 70 years at the time of enrollment.

### 2.3 Exclusion Criteria

Patients were excluded from the study based on the following criteria:

1. Presence of distant metastases (M1 disease) at diagnosis.
2. History of any other active malignancy within the past 5 years, except for adequately treated basal cell carcinoma of the skin or carcinoma *in situ* of the cervix.
3. Severe, uncontrolled comorbid conditions (e.g., uncompensated heart failure, severe chronic obstructive pulmonary disease, or active systemic infection).
4. Pregnancy or lactation.

### 2.4 Pre-Treatment Evaluation and Sample Collection

The initial evaluation included a comprehensive diagnostic workup to determine disease extent and severity. This included a thorough physical examination, complete blood cell count, routine serum chemistry panels, creatinine clearance assessment, chest X-ray, computed tomography (CT) or magnetic resonance imaging (MRI) of the head and neck, and a bone scan. Additionally, triple endoscopy was conducted to evaluate local tumor extent and regional metastases. During the endoscopic procedure, biopsies were obtained from both the tumor and adjacent normal tissues for histopathological and molecular analysis. Blood samples were collected from all enrolled patients and healthy control subjects using standard venipuncture techniques. Serum was separated by centrifugation at 3000× *g* for 15 min at 4°C within 2 h of collection and aliquoted. All serum samples were visually inspected for hemolysis, and those showing visible pink or red discoloration were excluded from further analysis. The remaining serum was stored at −80°C for subsequent RNA extraction.

### 2.5 Treatment Regimen

The induction chemotherapy regimen consisted of three cycles of docetaxel (75 mg/m^2^ on day 1) combined with cisplatin (75 mg/m^2^ on day 1), along with a continuous intravenous infusion of fluorouracil (500 mg/m^2^ per day) from days 1 to 5. This cycle was repeated every four weeks.

### 2.6 Treatment Response Assessment and Subsequent Management

Patient response to induction chemotherapy was assessed prior to each subsequent cycle through clinical and radiological evaluations. Clinical assessments included endoscopic inspection and palpation of lymph nodes. To evaluate tumor response radiologically, CT or MRI scans were performed after the second chemotherapy cycle. Treatment response was categorized as follows: a Complete Response (CR) was defined as the disappearance of all target lesions; a Partial Response (PR) was defined as at least a 30% decrease in the sum of diameters of target lesions, taking as reference the baseline sum diameters; Stable Disease (SD) was defined as neither sufficient shrinkage to qualify for PR nor sufficient increase to qualify for progressive disease; and Progressive Disease (PD) was defined as at least a 20% increase in the sum of diameters of target lesions, taking as reference the smallest sum on study, or the appearance of one or more new lesions [20]. Patients achieving a complete response (CR) or partial response (PR) received a maximum of three chemotherapy cycles before proceeding to definitive radiation therapy. In cases of disease progression, surgical resection followed by radiation therapy was performed. Patients who achieved CR or PR after the third chemotherapy cycle advanced to radiation therapy.

Radiation therapy was administered to all patients, either immediately following chemotherapy for responders or after salvage surgery for non-responders. The treatment targeted both sides of the neck and the primary tumor site, delivered in daily fractions of 2 Gy, five days per week. Patients receiving irradiation post-chemotherapy received a total dose of 5000 cGy, supplemented by an additional boost of 2000 cGy focused on the tumor site and any palpable lymph nodes. Following salvage surgery, a radiation dose of 5000 cGy was administered, with a booster dose of 14 Gy applied to areas with positive margins, extracapsular spread, or three or more involved lymph nodes.

Response to radiation therapy was re-evaluated twelve weeks after completion. Patients with persistent laryngeal disease underwent salvage laryngectomy. If the primary tumor was controlled but neck disease persisted, only neck dissection was performed. The extent of surgical resection was determined by the initial tumor size before chemotherapy. Classic wide-field total laryngectomy was performed for all primary tumors. Regional neck dissection was carried out on all surgical patients, with the exception of those with T3N0 or midline supraglottic T4N0 tumors where the risk of occult metastases to a specific neck side was unclear. The presence of residual primary tumors in patients undergoing salvage surgery was confirmed by biopsy.

All patients were managed under a standardized follow-up protocol with clinical examinations scheduled every three months for the first two years, every six months for the subsequent three years, and annually thereafter. Each visit entailed a comprehensive assessment, including a detailed history, physical examination with fiberoptic laryngoscopy, and annual radiological imaging of the head and neck for the first three years. The primary treatment response was definitively evaluated through histopathological confirmation via tissue biopsy after the completion of both neoadjuvant chemotherapy and definitive radiation therapy.

### 2.7 Quantitative Real-Time Polymerase Chain Reaction (qRT-PCR) for miR-449a

Total RNA, including microRNAs (miRNAs), was extracted from tumor tissue, adjacent normal tissues, and serum using TRIzol reagent (Thermo Fisher Scientific, Invitrogen, Cat. No. 15-596-018, Carlsbad, CA, USA) according to the manufacturer’s instructions. cDNA synthesis was performed using the Transcript First-Strand Synthesis Supermix (TransGen Biotech, Supermix AT301, Beijing, China.) as per the manufacturer’s protocol. qRT-PCR was performed using TaKaRa reagents (Takara Bio Inc., Cat. #R011, Kusatsu, Japan) on an Applied Biosystems 7900 Fast Real-Time PCR System. U6 was utilized as the internal control. The use of U6 was validated for this specific cohort by demonstrating a strong correlation (Pearson r = 0.94, *p* < 0.0001) between serum miR-449a expression levels normalized to U6 and those normalized using the global mean method, confirming the reported findings.

The primer sequences were as follows: miR-449a was amplified using a forward primer with the sequence 5^′^ → 3^′^ GCCGATGGCAGTGTATTGTTAG and a gene-specific reverse primer with the sequence 5^′^ → 3^′^ CTAACCAATACACTGCCATC. For the U6 small nuclear RNA, the forward primer sequence was 5^′^ → 3^′^ GGGTGCTCGCTTCGGCAGC, and the reverse primer was 5^′^ → 3^′^ CAGTGCAGGGTCCGAGGT.

Each sample was analyzed in triplicate. The expression of miR-449a was quantified as relative fold-change across sample groups using 2^−ΔΔCt^ [21].

The RT-PCR reaction mix had a total volume of 20 μL, comprising 10 μL of 2× Ultra SYBR one-step qRT-PCR Buffer, 2 μL of RNA template, 1 μL each of forward and reverse primers, 0.5 μL of Super enzyme mix, and 5.5 μL of nuclease-free water. The thermocycling conditions were: pre-denaturation at 95°C for 10 min, followed by 40 cycles of denaturation at 95°C for 15 s, and annealing/extension at 60°C for 1 min.

### 2.8 Statistical Analysis

Data analysis was performed using IBM SPSS Statistics software, version 27 (IBM SPSS Statistics, Armonk, NY, USA). Student’s *t*-test was used to compare means and standard deviations across groups. Receiver operating characteristic (ROC) curve analysis was conducted for both tissue and serum samples to assess the diagnostic accuracy of *miR-449a*. Kaplan-Meier curves were utilized to estimate the survival rates of LA-LSCC patients, and differences in survival outcomes were evaluated using the log-rank test. Further statistical methods included multiple regression analysis to assess the impact of clinical and pathological variables on miR-449a expression, and Cox proportional hazards regression analysis to identify independent predictors of overall survival. A Bonferroni correction was applied to account for multiple comparisons in the regression models. The significance threshold was set at *α* = 0.05, and *p*-values were adjusted accordingly. A Bonferroni-adjusted *p*-value of <0.05 was considered statistically significant for these analyses.

## 3 Results

### 3.1 Distribution of Nodal Involvement by Tumor Stage in Laryngeal Squamous Cell Carcinoma

The distribution of nodal stage (N-stage) in relation to tumor stage (T-stage) among patients with LSCC reveals a clear pattern of increasing cervical lymph node involvement with advancing primary tumor stage. Notably, N2 disease is more frequently observed in T4 tumors, underscoring the association between greater tumor burden and regional metastasis (Table 1).

**Table 1:** Distribution of nodal stage (N-stage) and tumor stage (T-stage) in patients with locally advanced laryngeal squamous cell carcinoma (LA-LSCC)

T-Stage	Nodal Stage N0 (*n*)	Nodal Stage N1 (*n*)	Nodal Stage N2 (*n*)	Total (*n*)
T2	0	0	11	11
T3	12	26	7	45
T4	9	10	6	25
Total (*n*)	21	36	24	81

Note: *n*, sample size.

### 3.2 Clinical Response to Induction Chemotherapy and Radiation Therapy in LA-LSCC

Table 2 presents the clinical response to induction chemotherapy (IC) followed by radiation therapy (RT) in patients with locally advanced laryngeal squamous cell carcinoma. Induction chemotherapy resulted in aCR in 31% of the 81 patients and a PR in 49%, with 20% exhibiting SD. Among the 65 patients who subsequently received RT, the complete response rate significantly increased to 74%, while 20% achieved PR and 6% SD. These data suggest that while IC provides initial tumor control in a notable proportion of patients, the addition of RT substantially enhances the rate of complete remission, indicating a synergistic effect of this sequential treatment strategy.

**Table 2:** Clinical response to induction chemotherapy and radiation therapy in patients with LSCC

Treatment modality	Number of patients (*n*)	Response type	Number (%)
After induction chemotherapy	81	Complete response	25 (31%)
		Partial response	40 (49%)
		Stable disease	16 (20%)
After radiation therapy	65	Complete response	48 (74%)
		Partial response	13 (20%)
		Stable disease	4 (6%)

### 3.3 Surgical Salvage Strategies in Locally Advanced Laryngeal Squamous Cell Carcinoma Following Induction Chemotherapy or Chemo-Radiation Therapy

The surgical salvage strategies employed following initial treatment are detailed in Table 3. After induction chemotherapy (IC), 16 patients required salvage surgery. The most extensive procedure, total laryngectomy (with or without partial pharyngectomy), was performed in 14 patients, while organ-preserving hemi-laryngectomy was pursued in 7 patients. The majority of these patients (14 of 16, 87.5%) underwent a concurrent neck dissection (unilateral or bilateral).

**Table 3:** Surgical salvage strategies in LA-LSCC following induction chemotherapy and radiation therapy

Salvage strategy & procedure	Unilateral neck dissection	Bilateral neck dissection	No neck dissection	Procedure total (*n*)
After induction chemotherapy (*n* = 16)				
Total laryngectomy + Partial pharyngectomy	4	—	—	4
Total laryngectomy	5	3	2	10
Hemi laryngectomy	4	3	—	7
After radiation therapy (*n* = 17)				
Hemi laryngectomy	4	1	3	8
Neck dissection only	8	5	—	13
Total, *n* (%)	25 (76%)	12 (36%)	5 (15%)	33

Note: Some patients underwent more than one procedure.

In contrast, salvage surgery after radiation therapy (RT) was characterized by a less invasive approach, primarily focused on managing residual nodal disease. Among the 17 patients in this group, the most common procedure was neck dissection alone (13 of 17 patients, 76.5%), with only 4 patients requiring a hemi-laryngectomy. Overall, unilateral neck dissection was the most frequent approach across all salvage procedures, performed in 25 of 33 total cases (75.8%).

### 3.4 miR-449a Expression and Diagnostic Accuracy in Laryngeal Squamous Cell Carcinoma: Insights from Tissue and Serum Analysis

**Figure 1:**
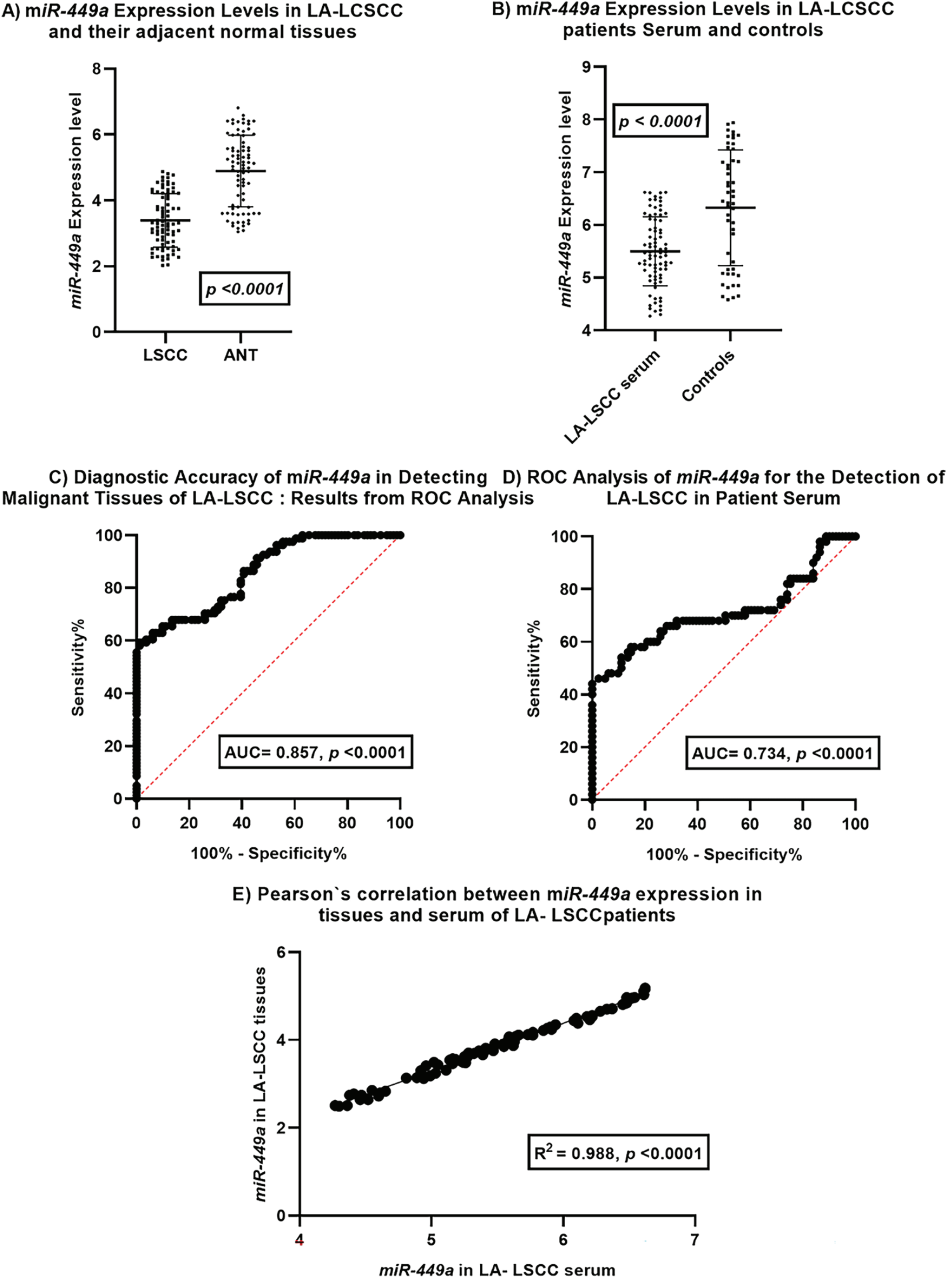
*miR-449a* Expression and Diagnostic Potential in Laryngeal Squamous Cell Carcinoma. This figure illustrates the expression patterns and diagnostic utility of *miR-449a* in laryngeal squamous cell carcinoma (LSCC).(**A**) *miR-449a* expression levels are significantly downregulated in LSCC tissues (*n* = 81) compared to adjacent normal tissues (ANT, *n* = 81). (**B**) Serum *miR-449a* levels are also significantly lower in LSCC patients (*n* = 81) compared to healthy controls (*n* = 50). (**C**) ROC curve analysis demonstrates good diagnostic accuracy of tissue *miR-449a* in distinguishing malignant from normal laryngeal tissue (AUC = 0.857, *p* < 0.0001; *n* = 81 pairs). (**D**) ROC curve analysis reveals moderate diagnostic accuracy of serum miR-449a for LSCC detection in patient serum (AUC = 0.734, *p* < 0.0001; patients *n* = 81 pairs, controls *n* = 50). (**E**) A strong positive correlation is observed between *miR-449a* expression levels in LSCC tissues and corresponding patient serum samples (R^2^ = 0.988, *p* < 0.0001; *n* = 81 matched pairs). Error bars in (**A**) and (**B**) represent standard deviation

Analysis of miR-449a expression in laryngeal squamous cell carcinoma (LSCC) tissues, adjacent normal laryngeal tissues (ANT), and serum, along with its diagnostic potential (Fig. 1).

Panel A compares *miR-449a* expression levels in LSCC tissues and their corresponding adjacent normal tissues. The dot plot with error bars indicates a statistically significant downregulation of *miR-449a* in LSCC tissues compared to ANT (*p* < 0.0001). This suggests a potential tumor-suppressive role for *miR-449a* in LSCC development.

Panel B compares serum *miR-449a* expression levels in LSCC patients and healthy controls. The dot plot with error bars reveals a statistically significant downregulation of miR-449a in the serum of LSCC patients compared to controls (*p* < 0.0001). This observation suggests the potential utility of serum *miR-449a* as a diagnostic biomarker.

Panel C displays the Receiver Operating Characteristic (ROC) curve for *miR-449a* in distinguishing malignant LSCC tissues from adjacent normal laryngeal tissues. The Area Under the Curve (AUC) of 0.857 with a highly significant *p*-value (<0.0001) indicates good diagnostic accuracy of tissue miR-449a in identifying malignant tissue.

Panel D presents the ROC curve for serum *miR-449a* in detecting LSCC in patient serum. The AUC of 0.734 with a highly significant *p*-value (<0.0001) demonstrates moderate diagnostic accuracy of serum *miR-449a* for LSCC detection. While lower than the AUC for tissue, it still suggests potential clinical utility as a less invasive biomarker.

Panel E investigates the correlation between *miR-449a* expression levels in tissues and serum of LSCC patients. The scatter plot shows a strong positive correlation (R^2^ = 0.988, *p* < 0.0001) between *miR-449a* levels in LSCC tissues and their corresponding serum samples. This strong correlation suggests that serum miR-449a levels may reflect tissue expression levels in LSCC patients, supporting its potential as a surrogate biomarker.

In summary, this figure provides evidence for the significant downregulation of *miR-449a* in both LSCC tissues and patient serum compared to their respective controls. Both tissue and serum *miR-449a* demonstrate statistically significant diagnostic accuracy for LSCC, with tissue showing better performance. Importantly, the strong positive correlation between tissue and serum *miR-449a* levels in LSCC patients supports the potential of serum *miR-449a* as a non-invasive biomarker that reflects tumor expression. These findings warrant further investigation for clinical translation.

### 3.5 Independent Clinicopathological Correlates of miR-449a Expression in Laryngeal Squamous Cell Carcinoma

Univariate analysis revealed that higher miR-449a expression was significantly associated with all tested clinicopathological parameters (Table 4; all *p* < 0.0001). Elevated expression was correlated with older age (46–69 years), female sex, non-smoking status, and less aggressive disease, specifically lower tumor grade (I–II), early T-stage (T1–T2), limited nodal involvement (N0–N1), and earlier TNM stage (Stage III).

**Table 4:** Association between *miR-449a* expression levels and clinicopathological features in patients with LA-LSCC

Variable	Category	Number (N)	*miR-449a* (Mean ± SD)	Effect size (Cohen’s d)	t-Value	*p*-Value
Age group	35–45 years	39	2.696 ± 0.365	3.17	14.09	<0.0001
	46–69 years	42	4.049 ± 0.491			
Sex	Male	63	3.069 ± 0.606	3.03	9.923	<0.0001
	Female	18	4.513 ± 0.199			
Smoking status	Smoker	57	2.965 ± 0.515	3.49	13.37	<0.0001
	Non-smoker	24	4.424 ± 0.235			
Tumor grade	I–II	38	3.636 ± 0.688	2.52	7.391	<0.0001
	III–IV	43	2.309 ± 0.153			
T-stage	T1–T2	11	3.57 ± 0.719	2.52	6.078	<0.0001
	T3–T4	70	2.243 ± 0.119			
N-stage	N0–N1	24	3.79 ± 0.608	2.83	10.55	<0.0001
	N2–N3	57	2.44 ± 0.219			
TNM stage	Stage III	38	4.03 ± 0.501	3.15	14.02	<0.0001
	Stage IV	43	2.67 ± 0.350			

The calculated effect sizes (Cohen’s d) were notably large (d > 3.0) for several comparisons, including smoking status and TNM stage, indicating a substantial and clinically relevant difference in miR-449a expression between these groups. The notably large effect sizes observed are likely attributable to the highly distinct biological states being compared (e.g., low-grade vs. high-grade tumors) and the pronounced downregulation of *miR-449a* in aggressive disease subtypes.

These univariate findings suggest a complex relationship between *miR-449a* expression and patient profile, where higher expression is linked to both favorable disease characteristics and older age. To identify which factors are independent predictors, a multivariate regression analysis was performed (Table 5). This subsequent analysis confirmed that higher *miR-449a* expression was independently associated with female sex (*p* = 0.004), non-smoking status (*p* < 0.0001), and older age (*p* = 0.004). Conversely, more advanced disease—defined by higher T-stage (*p* = 0.004), N-stage (*p* < 0.001), and TNM stage (*p* = 0.001)—was independently associated with significantly lower miR-449a expression. Notably, while tumor grade and performance status showed strong univariate associations, they were not found to be independent predictors in the multivariate model (*p* = 0.197 and *p* = 0.066, respectively).

**Table 5:** Analyzing the impact of clinical and pathological variables on *miR-449a* Expression: a multiple regression study in LA-LSCC

Variable	Category/Definition	Estimate	Standard error	95% CI	t-Value	*p*-Value
Intercept		3.205	0.512	2.179 to 4.231	6.258	<0.0001
Sex	Female vs. Male	0.512	0.175	0.101 to 0.863	2.925	0.004
Age	Older vs. Younger	0.075	0.025	0.025 to 0.125	3	0.004
Smoking status	Non-Smoker vs. Smoker	0.921	0.201	0.517 to 1.325	4.578	<0.0001
Tumor grade	III-IV vs. I-II	0.334	0.257	−0.180 to 0.848	1.299	0.197
T-stage	T3-T4 vs. T1-T2	−0.498	0.15	−0.838 to −0.158	−2.929	0.004
N-stage	N2-N3 vs. N0-N1	−0.663	0.15	−0.963 to −0.363	−4.420	<0.0001
TNM stage	Stage IV vs. Stage III	−0.8	0.23	−1.260 to −0.340	−3.478	0.001
Performance status	ECOG 1–2 vs. 0	0.326	0.175	−0.025 to 0.677	1.863	0.066
Treatment response	Responder vs. non-responder	0.512	0.175	0.161 to 0.863	2.925	0.004

The highly significant univariate associations support *miR-449a’*s potential utility as a biomarker, while the multivariate analysis refines our understanding by identifying older age, female sex, non-smoking status, and less advanced disease as the key independent factors associated with its elevated expression.

### 3.6 miR-449a Expression Dynamics as a Potential Indicator of Treatment Efficacy in Laryngeal Cancer

Table 6 presents the change in *miR-449a* expression levels in relation to treatment response following induction chemotherapy and radiation therapy in patients with laryngeal squamous cell carcinoma (LSCC). Paired *t*-tests were utilized to assess the significance of the change in *miR-449a* expression within each response group.

**Table 6:** *miR-449a* expression changes with treatment response in LA-LSCC

Treatment modality	Response type	No.	*miR-449a* (Before)	*miR-449a* (After)	t-Value	*p*-Value
Induction Chemotherapy	Complete response (CR)	25	2.369 ± 0.416	3.948 ± 0.491	12.27	<0.0001
	Partial response (PR)	40	2.728 ± 0.360	3.984 ± 0.447	13.85	<0.0001
Radiation therapy	Complete response (CR)	48	2.809 ± 0.421	3.472 ± 0.401	7.008	<0.0001
	Partial response (PR)	13	2.170 ± 0.353	3.482 ± 0.740	5.77	<0.0001

Note: No.: sample size.

Following induction chemotherapy, patients who achieved a Complete Response (CR, *n* = 25) exhibited a significant increase in *miR-449a* expression from a mean of 2.369 ± 0.416 to 3.948 ± 0.491 (*p* < 0.0001). Similarly, patients with a Partial Response (PR, *n* = 40) to induction chemotherapy also showed a significant increase in miR-449a expression from 2.728 ± 0.360 to 3.984 ± 0.447 (*p* < 0.0001).

In the subsequent radiation therapy phase, patients who achieved a Complete Response (CR, *n* = 48) demonstrated a significant increase in *miR-449a* expression from a mean of 2.809 ± 0.421 to 3.472 ± 0.401 (*p* < 0.0001). Likewise, patients with a Partial Response (PR, *n* = 13) to radiation therapy also showed a significant increase in *miR-449a* expression from 2.170 ± 0.353 to 3.482 ± 0.740 (*p* < 0.0001).

This suggests a potential association between increased *miR-449a* expression and favorable tumor response to these treatment modalities in LSCC. Further investigation is warranted to elucidate the underlying mechanisms and the potential role of *miR-449a* as a predictive marker for treatment response.

### 3.7 miR-449a and Clinical Outcome in LA-LSCC: Insights from Survival Analysis

**Figure 2:**
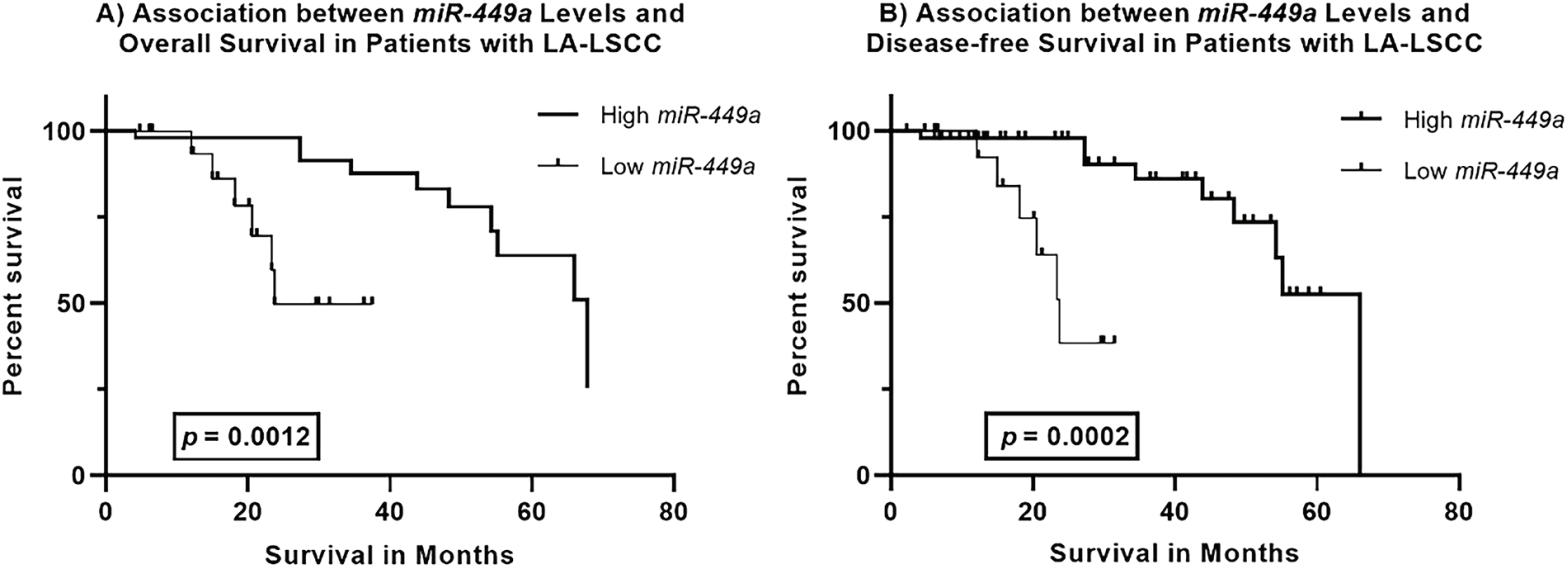
Kaplan–Meier Survival Curves for *miR-449a* Expression in Locally Advanced Laryngeal Squamous Cell Carcinoma (LA-LSCC). (**A**). Association between *miR-449a* Levels and Overall Survival in LA-LSCC Patients Kaplan–Meier survival curves comparing overall survival in patients with locally advanced laryngeal squamous cell carcinoma (LA-LSCC), stratified by *miR-449a* expression levels. Patients with high *miR-449a* expression (solid line) demonstrated significantly longer overall survival compared to those with low *miR-449a* expression (dashed line; Log-rank test, *p* = 0.0012). The median overall survival was 67.82 months in the high *miR-449a* group vs. 23.74 months in the low *miR-449a* group. (**B**). Association between *miR-449a* Levels and Disease-Free Survival in LA-LSCC Patients Kaplan–Meier survival curves comparing disease-free survival in LA-LSCC patients stratified by miR-449a expression levels. Patients with high *miR-449a* expression (solid line) exhibited significantly prolonged disease-free survival compared to those with low miR-449a expression (dashed line; Log-rank test, *p* = 0.0002). The median disease-free survival was 66.03 months in the high *miR-449a* group vs. 23.74 months in the low *miR-449a* group

Kaplan-Meier survival analysis reveals a significant association between *miR-449a* expression levels and survival outcomes in Locally Advanced Laryngeal Squamous Cell Carcinoma (LA-LSCC) patients (Fig. 2).

***Overall Survival:*** Patients with high *miR-449a* expression demonstrated a significantly longer overall survival compared to those with low expression (Log-rank test, *p* = 0.0012). The median overall survival was 67.82 months in the high *miR-449a* group vs. 23.74 months in the low *miR-449a* group.

***Disease-Free Survival:*** Similarly, high *miR-449a* expression was associated with significantly prolonged disease-free survival (Log-rank test, *p* = 0.0002). The median disease-free survival was 66.03 months for patients with high *miR-449a* expression, compared to 23.74 months for those with low expression.

These findings indicate that higher *miR-449a* expression is a strong predictor of both improved overall survival and prolonged disease-free survival in LA-LSCC patients, suggesting its potential as a favorable prognostic biomarker for this malignancy.

### 3.8 Prognostic Significance of miR-449a Expression in Locally Advanced Laryngeal Squamous Cell Carcinoma: A Multivariate Survival Analysis

The Cox proportional hazards regression analysis presented in Table 7 identifies *miR-449a* expression and several clinicopathological variables as independent predictors of overall survival in patients with locally advanced laryngeal squamous cell carcinoma (LA-LSCC). High *miR-449a* expression was significantly associated with a reduced risk of death (HR = 0.15, 95% CI: 0.055–0.410, *p* < 0.001), suggesting a protective role in survival outcomes. In contrast, older age (HR = 1.043, 95% CI: 1.019–1.068, *p* < 0.001), higher tumor grade (HR = 3.434, 95% CI: 1.630–7.236, *p* = 0.001), advanced T-stage (HR = 4.794, 95% CI: 2.024–11.358, *p* < 0.001), and higher TNM stage (HR = 4.29, 95% CI: 1.996–9.223, *p* < 0.001) were all independently linked to an increased hazard of mortality. Associations for smoking status and N-stage were observed but did not retain statistical significance after correction for multiple comparisons. These findings highlight the robust prognostic value of *miR-449a* alongside key established clinical factors in stratifying patient risk.

**Table 7:** Multivariate cox regression analysis of survival in patients with LA-LSCC

Variable	Category	Coefficient (β)	Standard error	Hazard ratio (HR)	95% CI for HR	*p*-Value	Significance
miR-449a Expression	High vs. Low	−1.897	0.512	0.15	0.055–0.410	<0.001	***
Age	Continuous	0.042	0.012	1.043	1.019–1.068	<0.001	***
Sex	Male vs. Female	0.652	0.398	1.919	0.880–4.184	0.102	NS
Smoking status	Smoker vs. Non-smoker	0.876	0.367	2.401	1.168–4.936	0.017	*
Tumor grade	III–IV vs. I–II	1.234	0.38	3.434	1.630–7.236	0.001	**
T-stage	T3–T4 vs. T1–T2	1.567	0.44	4.794	2.024–11.358	<0.001	***
N-stage	N2–N3 vs. N0–N1	1.102	0.398	3.01	1.380–6.568	0.006	**
TNM stage	Stage IV vs. Stage III	1.456	0.39	4.29	1.996–9.223	<0.001	***

Note: Statistically significant, *p < 0.05; **p < 0.01; ***p < 0.001; NS: Non-Significant.

## 4 Discussion

The management of locally advanced laryngeal squamous cell carcinoma (LA-LSCC) has increasingly shifted toward multimodal strategies that integrate surgery, radiotherapy, chemotherapy, and targeted or immunotherapeutic agents to improve patient outcomes [22]. For advanced-stage disease, concurrent chemoradiation has become a standard of care, aimed at reducing tumor burden, eradicating micrometastatic disease, and preserving laryngeal function—thereby significantly enhancing quality of life [23].

Our results demonstrate the efficacy of a sequential treatment strategy for LA-LSCC. The marked increase in the complete response rate from 31% after induction chemotherapy to 74% following subsequent radiotherapy underscores a clear synergistic effect between these modalities. This approach successfully enabled laryngeal preservation in the majority of patients, thereby preserving critical functions and improving quality of life. These findings support the use of induction chemotherapy followed by radiotherapy for LA-LSCC and are consistent with other studies [24–27].

The optimization of treatment strategies for LA-LSCC requires novel biomarkers for early diagnosis, accurate prognosis, and prediction of therapeutic response. This study was designed to investigate microRNA-449a (*miR-449a*) as such a biomarker. We aimed to evaluate its diagnostic potential by comparing expression profiles in LA-LSCC patients vs. healthy controls, and to assess its prognostic value by correlating expression levels with key clinicopathological features and survival outcomes. Through a comprehensive analysis, this research sought to validate *miR-449a* as a non-invasive biomarker and determine its utility for improving risk stratification and personalizing treatment strategies.

Our findings establish *miR-449a* as a promising diagnostic biomarker for LA-LSCC. We observed significant downregulation of miR-449a in tumor tissues compared to adjacent normal mucosa (*p* < 0.0001). This aligns with its well-documented tumor-suppressor role in other cancers [12,14,28] and is consistent with reports of its downregulation in HNSCC [14] and specifically in LSCC [17]. The recent work by Cossu et al. provides a mechanistic basis for our findings, showing that miR-449a antagonizes EMT in LSCC [17]. Our results confirm the critical clinical relevance of this biological role, as low *miR-449a* was independently associated with advanced T-stage and N-stage, hallmarks of invasive and metastatic disease.

This downregulation is a hallmark of carcinogenesis and can be mechanistically explained by several factors. Firstly, epigenetic silencing, particularly hypermethylation of the promoter region of the *miR-449a* host gene, is a common mechanism that suppresses its transcription. Secondly, chromosomal deletions or loss of heterozygosity at its genomic locus (5q11.2) may contribute to its loss. Functionally, the silencing of *miR-449a* provides a strong selective advantage for cancer cells. *miR-449a* acts as a key regulator of cell cycle progression, differentiation, and apoptosis by directly targeting and repressing critical oncogenes and cell cycle promoters, such as CDK6, MET, and NOTCH1. Consequently, the loss of *miR-449a* expression in LA-LSCC releases the brakes on proliferation, disrupts apoptotic pathways, and can promote epithelial-to-mesenchymal transition, thereby driving tumor initiation, progression, and aggressive behavior [29].

This pattern was replicated in a non-invasive context, with serum levels also significantly lower in patients than in healthy controls (*p* < 0.0001). ROC curve analysis confirmed strong diagnostic accuracy for tissue *miR-449a* (AUC = 0.857, *p* < 0.0001), while serum miR-449a showed moderate accuracy (AUC = 0.734, *p* < 0.0001). The strong positive correlation between tissue and serum miR-449a levels (R^2^ = 0.988, *p* < 0.0001) indicates that circulating *miR-449a* reliably reflects intratumoral expression, supporting its utility as a non-invasive surrogate biomarker. These results align with the growing role of microRNAs as diagnostic tools in head and neck cancers. To our knowledge, this is the first study to comprehensively validate the dual-tissue and serum-based diagnostic potential of *miR-449a* specifically in a well-defined cohort of LA-LSCC, thereby highlighting its promise as a non-invasive biomarker.

We evaluated the relationship between *miR-449a* expression and key clinicopathological parameters. Univariate analysis revealed that high *miR-449a* expression was significantly associated with older age, female sex, non-smoking status, and less aggressive disease, characterized by lower tumor grade, earlier T-stage, and earlier TNM stage (all *p* < 0.0001).

Multivariate analysis confirmed that advanced disease stage was independently associated with reduced *miR-449a* expression, specifically higher T-stage (*p* = 0.004), N-stage (*p* < 0.0001), and TNM stage (*p* = 0.001). Female sex (*p* = 0.004) and older age (*p* = 0.004) were independent predictors of higher expression, while smoking was a significant negative predictor (*p* < 0.0001). Pathological grade and performance status were not independently associated.

The consistent inverse association between *miR-449a* and disease stage across both analyses reinforces its role as a tumor suppressor in LA-LSCC, whose loss promotes aggressive tumor behavior. The independent link with older age represents a novel finding that warrants further investigation.

Beyond its diagnostic potential, our study establishes *miR-449a* as a significant independent prognostic factor in LA-LSCC. Kaplan-Meier analysis revealed that patients with high *miR-449a* expression had significantly prolonged overall survival (median 67.82 vs. 23.74 months; Log-rank *p* = 0.0012) and disease-free survival (median 66.03 vs. 23.74 months; Log-rank *p* = 0.0002). Multivariate Cox regression confirmed high *miR-449a* expression as an independent predictor of improved overall survival (Hazard Ratio [HR] = 0.15, 95% CI: 0.055–0.410, *p* < 0.001), indicating a strong protective effect. These findings align with previous studies reporting miR-449a as a predictor of favorable cancer-specific survival in other malignancies [9,12,30].

Our multivariate analysis also confirmed established prognostic factors, with older age (*p* < 0.001), higher tumor grade (*p* = 0.001), advanced T-stage (*p* < 0.001), and TNM stage (*p* < 0.001) all independently predicting an increased hazard of mortality. The associations for smoking status and N-stage did not retain independent significance after rigorous adjustment for multiple comparisons.

A nuanced finding from our multivariate analysis warrants emphasis: although high *miR-449a* expression was an independent predictor of improved survival, advanced age was a powerful independent predictor of worse outcomes (HR = 1.043 per year, *p* < 0.001). This apparent divergence reflects the complex biology of LA-LSCC, where advanced age—a well-established adverse prognostic factor linked to comorbidities, reduced treatment tolerance, and distinct tumor biology—confers a baseline increased risk. The persistence of *miR-449a’*s powerful protective association after adjusting for age and other key factors solidifies its validity as a robust biomarker. This suggests that among older patients, who generally have a poorer prognosis, those with high *miR-449a* expression represent a biological subgroup with more favorable disease. Consequently, *miR-449a* could be particularly valuable for refining risk stratification in older LA-LSCC patients, potentially identifying those most likely to tolerate and benefit from intensive curative-intent therapy.

The robust independent prognostic value of *miR-449a* indicates its potential for refining risk stratification in LA-LSCC. Our data, which corroborate the findings of Cossu et al. [17], The robust independent prognostic value of *miR-449a* indicates its potential for refining risk stratification in LA-LSCC. Our data, which corroborate the findings of Cossu et al. Moreover, our study provides a novel translational dimension by establishing that serum *miR-449a* levels strongly correlate with tissue expression and exhibit dynamic changes during treatment. This position circulating *miR-449a* as a practical tool for non-invasive risk stratification and response monitoring, moving beyond tissue-based analysis.

We also assessed *miR-449a* expression dynamics during treatment to evaluate its role as a predictor of therapeutic response. In patients who achieved a complete or partial response, *miR-449a* levels increased significantly following both induction chemotherapy and subsequent radiotherapy (all *p* < 0.0001). For example, in complete responders to induction chemotherapy, levels rose from 2.369 ± 0.416 to 3.948 ± 0.491. A similar increase was observed in complete responders to radiotherapy, from 2.809 ± 0.421 to 3.472 ± 0.401. These dynamic changes suggest that effective treatment—through mechanisms such as tumor cell apoptosis or reduced tumor burden—induces a rebound in *miR-449a* expression. This positions *miR-449a* as a promising real-time, circulating biomarker for monitoring therapeutic efficacy, potentially enabling adaptive treatment strategies. Similar *miR-449a* dynamics have been reported in other malignancies; for instance, elevated post-operative levels were associated with treatment response in osteosarcoma, supporting its role in evaluating therapeutic success [31].

Several limitations of this study should be acknowledged. The single-center design and cohort size of 81 patients, while sufficient for initial analyses, may affect the generalizability of our findings, particularly given the potential for ethnic and regional variations in miRNA expression and disease biology. Future validation in larger, multi-institutional, and multi-ethnic prospective cohorts, utilizing standardized pre-analytical and analytical protocols, is essential to confirm the robust clinical utility of *miR-449a* and to establish its prognostic value across diverse patient populations.

Furthermore, the precise molecular mechanisms through which *miR-449a* exerts its tumor-suppressive effects in LA-LSCC remain to be fully elucidated. Future work should aim to identify its direct mRNA targets and the downstream pathways governing cell cycle, apoptosis, and epithelial-mesenchymal transition. Functional studies, including *miR-449a* overexpression and knockdown in LSCC models, are needed to establish a causal link between *miR-449a* expression and tumor phenotype.

The translation of *miR-449a* into a routine clinical biomarker also faces several practical challenges. These include the need for standardized protocols for sample collection (e.g., tube type, time-to-processing, and hemolysis assessment), RNA extraction, and data normalization to ensure reproducibility and comparability across different laboratories. The scalability of qRT-PCR assays for high-throughput clinical use and the establishment of universally accepted reference ranges for serum *miR-449a* levels are also significant hurdles that must be addressed. Finally, integrating *miR-449a* into multiparametric biomarker panels may enhance its diagnostic and prognostic performance and improve its cost-effectiveness for widespread clinical application.

## 5 Conclusions

In summary, our findings strongly support *miR-449a* as a promising novel biomarker for locally advanced laryngeal squamous cell carcinoma. Its significant downregulation in both tissue and serum indicates diagnostic potential, and its independent association with improved survival underscores its prognostic value. Moreover, the rebound in *miR-449a* expression following successful chemotherapy and radiotherapy positions it as a dynamic indicator of treatment response. Collectively, *miR-449a* emerges as a multi-faceted tool that could enhance non-invasive diagnosis, refine prognostic stratification, and enable response monitoring in LA-LSCC, thereby contributing to improved patient outcomes and laryngeal preservation.

## Data Availability

The data that support the findings of this study are available from the corresponding author, [Wael H. Elsawy], upon reasonable request.
